# Spatial immune ecosystems and therapy resistance in large B-cell lymphoma: from single-cell multi-omics to immunotherapy-matched validation

**DOI:** 10.3389/fimmu.2026.1916056

**Published:** 2026-10-01

**Authors:** Gaojie Liu, Junwei Yang, Han Li, Lishuo Deng, Liuxin Jiang, Yuetong Tan, Jianxiong Zhu, Jifan Li, Yao Wang

**Affiliations:** 1 Third Ward of Gastrointestinal Surgery, The Affiliated Cancer Hospital, Guangzhou Medical University, Guangzhou, Guangdong, China; 2 Medical Oncology Department, the Affiliated Cancer Hospital, Guangzhou Medical University, Guangzhou, Guangdong, China; 3 Guangzhou Institute of Cancer Research, The Affiliated Cancer Hospital, Guangzhou Medical University, Guangzhou, Guangdong, China

**Keywords:** antibody-drug conjugates, bispecific antibodies, cancer immunotherapy, CAR-T cells, diffuse large B-cell lymphoma, large B-cell lymphoma, pathology-ready biomarkers, spatial immunology

## Abstract

Large B-cell lymphomas, particularly diffuse large B-cell lymphoma (DLBCL), provide a clinically important model in which tumor-intrinsic programs, immune recognition, effector-cell fitness, and tissue architecture jointly shape response and resistance to immunotherapy. Although molecular classification has refined the understanding of lymphoma biology, relapse after chemoimmunotherapy, chimeric antigen receptor (CAR) T-cell therapy, CD20×CD3 bispecific antibodies, antibody-drug conjugates (ADCs), and immune-combination strategies cannot be explained by genetics or target-antigen expression alone. Single-cell and spatial multi-omics now allow lymphoma to be interpreted as a spatial immune ecosystem composed of malignant B-cell states, exhausted or regulatory T-cell programs, myeloid suppressive hubs, stromal and vascular compartmentalization, metabolic gradients, and local communication networks. This Review synthesizes recent DLBCL and large B-cell lymphoma ecosystem studies from a cancer-immunology perspective, emphasizing how spatial immune organization may influence effector access, antigen engagement, macrophage-mediated suppression, antibody exposure, and treatment-associated spatial change. We propose a treatment-matched validation framework that connects high-dimensional discovery to formalin-fixed paraffin-embedded (FFPE)-compatible pathology assays, including reduced marker panels for CAR T-cell therapy, bispecific antibodies, ADC-containing therapy, and immune-combination trials. Throughout, we distinguish observed tissue organization from computationally inferred communication and experimentally validated mechanisms. The resulting framework is intended as a trial-design and biomarker-development roadmap, not as a current clinical allocation algorithm.

## Highlights

- Large B-cell lymphoma therapy resistance is increasingly interpretable as a spatial cancer-immunity problem involving tumor-antigen expression, effector-cell access, macrophage-mediated suppression, and tissue architecture.
- Single-cell and spatial multi-omics support a niche-centered immunology framework, but most reported communication axes remain observational or computationally inferred rather than experimentally causal.
- Genotype-to-niche models, including antigen-presentation lesions, MCD-like inflammatory biology, and EZB-like immune-silent states, should be tested as site-aware hypotheses rather than deterministic rules.
- Spatial-pharmacology models for ADCs, CAR T cells, and bispecific antibodies should define what to measure prospectively, not current clinical allocation algorithms.
- Translation will require treatment-matched cohorts, reduced FFPE-compatible immune panels, coordinated spatial-data standards, and longitudinal validation of clinically meaningful spatial metrics.

## Introduction

Diffuse large B-cell lymphoma (DLBCL) is the most common aggressive lymphoma and remains clinically heterogeneous despite advances in molecular classification and therapy. R-CHOP remains an important backbone, while polatuzumab-containing frontline therapy, cellular therapy, CD20×CD3 bispecific antibodies, antibody-drug conjugates (ADCs), checkpoint-based approaches in selected contexts, and targeted combinations have broadened the immunotherapy and immune-engaging treatment landscape (1–6). Yet patients with apparently similar tumor-intrinsic features can experience very different outcomes, indicating that resistance cannot be explained by genetics or target-antigen expression alone (7–9).

Single-cell RNA sequencing, single-nucleus profiling, spatial transcriptomics, digital spatial profiling, imaging mass cytometry, multiplex immunofluorescence, and related assays now make it possible to study DLBCL as an interacting cancer-immune tissue ecosystem (10–25). These approaches separate malignant B-cell states from non-malignant immune and stromal compartments and, when spatial coordinates are preserved, reveal where immune cells are activated, exhausted, excluded, suppressed, or physically positioned relative to malignant cells. For pathology-facing translation, multiplex IHC/IF and high-plex imaging workflows provide a practical bridge from spatial discovery maps to FFPE-compatible immune-cell phenotyping, selection of regions of interest (ROIs), and digital image analysis (26–28).

This Review synthesizes high-resolution DLBCL and large B-cell lymphoma ecosystem studies into a cancer-immunology framework for immune escape and treatment resistance. It is not a systematic review or a pooled evidence count. Instead, it asks how malignant B-cell programs, T-cell dysfunction, macrophage and myeloid states, stromal or vascular compartments, and candidate communication axes can be translated into testable spatial biomarkers and treatment-matched validation studies for immune-engaging therapies. The strongest current evidence comes from convergent single-cell and spatial studies, including multimodal niche mapping in DLBCL, large B-cell lymphoma microenvironment archetype profiling, and myeloid-endothelial niche studies, while smaller or purely integrative analyses are treated as supporting or emerging evidence (22, 23, 25). This hierarchy keeps activated B-cell-like (ABC)-type integrative analyses in a hypothesis-generating role unless supported by treatment-annotated and coordinate-preserving validation (24).

Throughout this Review, DLBCL is used for studies restricted to diffuse large B-cell lymphoma, whereas large B-cell lymphoma (LBCL) is retained for cohorts that intentionally combine DLBCL with other large B-cell lymphoma entities or for pivotal trials enrolled under an LBCL definition. Evidence from primary central nervous system, testicular, EBV-positive, or other anatomically restricted DLBCL is treated as site-specific and is not generalized to conventional nodal or systemic DLBCL without within-site validation. **Tables 1**–**3** therefore identify the source population or patient setting for each representative study and trial.

**Table 1:** Nine representative high-resolution DLBCL/LBCL ecosystem studies and their evidence types.

Study	Disease context/material	Platform	Key ecosystem or niche finding	Treatment or resistance relevance	Evidence type/level	Caveat/validation need
Steen et al., 2021 (10), Cancer Cell	Large DLBCL transcriptomic cohorts with scRNA-seq-informed EcoTyper analysis	Bulk transcriptome deconvolution plus scRNA-seq-informed cell-state and ecosystem modeling	Defined recurrent malignant B-cell states and microenvironmental ecosystem configurations.	Provides a conceptual basis for interpreting prognosis and resistance as ecosystem-level phenomena.	Ecosystem modeling; orthogonal single-cell support; prognostic association; no native spatial coordinates.	Framework-level evidence; not a treatment-intervention study.
Ye et al., 2022 (11), Cell Reports	17 DLBCL tumors and 3 control samples; 94,324 cells	scRNA-seq	Mapped malignant B-cell, T-cell, myeloid, and immune compartments at single-cell resolution.	Shows intra- and inter-tumor heterogeneity relevant to immune escape.	Descriptive single-cell atlas; no native spatial coordinates or treatment-predictive validation.	Atlas-level evidence; treatment-response inference is indirect.
Colombo et al., 2022 (12), Blood Advances	33 DLBCL tissue cases	Imaging mass cytometry	Identified spatial subregions of immune activation, immune suppression, and immune exclusion.	Explains why immune abundance alone may not predict checkpoint biology or treatment response.	Coordinate-preserving spatial observation with clinicopathological and chemotherapy-response association; not predictive validation.	Spatial correlations require prospective validation.
Wright et al., 2023 (14), Blood Advances	51 *de novo* DLBCLs with triplicate sampling; 337,995 tumor and immune cells	27-plex high-parameter imaging	Defined immune-deficient, dendritic-cell-enriched, and macrophage-enriched tumor immune microenvironments.	Highlights spatial organization of immune engagement and macrophage-enriched contexts.	Coordinate-preserving spatial imaging and neighborhood classification; observational evidence without prospective prediction.	Imaging panel-dependent categories; functional validation remains limited.
Liu M et al., 2024 (15), Nature Communications	Reactive and malignant lymphoid tissues with DLBCL prognostic analyses	Spatial transcriptomics/whole-transcriptome spatial profiling	Revealed macrophage heterogeneity and macrophage-derived spatial signatures.	Links macrophage spatial states with COO subtype and overall survival.	Regional spatial observation with prognostic association and transcriptomic validation; not treatment-predictive.	Cross-platform macrophage annotation needs harmonization.
Dai L et al., 2024 (16), Biomarker Research	DLBCL single-cell/spatial cohorts with prognostic validation	scRNA-seq plus spatial transcriptomics	Linked high-glycolysis malignant B-cell states with TAM clusters and exhausted immune features.	Supports a metabolism-macrophage-exhaustion axis associated with poor prognosis.	Integrated single-cell/spatial observation with prognostic association; no causal or predictive validation.	Association does not prove glycolysis causes immune suppression.
Dai Y et al., 2025 (22), Nature Genetics	78 large B-cell lymphoma samples, predominantly DLBCL	Highly multiplexed spatial transcriptomics and proteomics/multimodal spatial profiling	Defined T-cell-enriched, plasma-cell-enriched, myeloid-enriched, stromal-enriched, tumor-B-cell-enriched, diffuse, and mixed niches.	Identifies targetable inflammatory niches and communication patterns relevant to immune therapy.	Multimodal coordinate-preserving spatial observation with orthogonal protein support; therapeutic implications remain hypothesis-generating.	Prospective treatment-specific validation is needed.
Li et al., 2025 (23), Cancer Cell	217 large B-cell lymphoma tumors in the main study framework	Single-nucleus multiome profiling plus bulk DNA/RNA integration	Defined lymphoma microenvironment archetype profiles with immune, stromal, fibroblast/macrophage, and tumor-rich patterns.	Provides outcome- and CD19 CAR T-cell-related microenvironmental stratification hypotheses.	Single-nucleus archetype profiling with outcome and CAR T-cell-associated subgroup evidence; not prospective treatment-interaction validation.	Large B-cell lymphoma-wide framework; DLBCL-specific implementation requires validation.
Ferrant et al., 2026 (25), Blood Advances	DLBCL, follicular lymphoma, and reactive secondary lymphoid organs; additional DLBCL validation cohorts	Mass cytometry, scRNA-seq, in silico analyses, IMC, and published MIBI-TOF validation	Identified DLBCL-enriched suppressive macrophages colocalized with blood endothelial cells and nominated an ANXA1–FPR1/2 vascular–myeloid axis.	Supports a vascular–myeloid suppressive niche associated with immunosuppression and adverse prognosis; motivates lineage- and vessel-aware validation.	Multimodal spatial observation; orthogonal imaging and functional support; prognostic association; not treatment-predictive.	Cross-disease, observational evidence; the proposed interaction and translational relevance require independent prospective clinical validation.

Evidence labels distinguish descriptive atlases, coordinate-preserving observations, orthogonal validation, prognostic associations, treatment-response associations, and prospective predictive evidence. None of the nine representative studies provides prospective treatment-interaction validation; treatment relevance is therefore hypothesis-generating. ABC, activated B-cell-like; CAR T-cell, chimeric antigen receptor T-cell; COO, cell of origin; DLBCL, diffuse large B-cell lymphoma; FL, follicular lymphoma; IMC, imaging mass cytometry; LBCL, large B-cell lymphoma; MIBI-TOF, multiplexed ion beam imaging by time of flight; scRNA-seq, single-cell RNA sequencing; TAM, tumor-associated macrophage.

**Table 2:** Spatial ecosystem modules and pathology-ready immunotherapy validation schema in DLBCL.

Resistance module	Pathology-ready spatial readout/proxy	Therapeutic relevance	Evidence boundary/validation priority
Malignant B-cell programs and antigen presentation	PAX5/CD79a-defined tumor-rich ROIs; CD20 retained as a target-antigen readout rather than the sole lineage anchor after anti-CD20 therapy; MHC-I/MHC-II and B2M/HLA status; tumor-state-enriched regions.	May shape immune recognition, CD8+ engagement, and sensitivity to chemoimmunotherapy or immune-based therapy.	Mostly associative; validate with matched genomics, spatial pathology, treatment annotation, and longitudinal sampling.
T-cell dysfunction and effector accessibility	Effector-cell accessibility: CD3+/CD8+ density within a tumor ROI plus, after segmentation QC, the fraction of lineage-defined tumor cells with an effector cell inside a locked radius. Tumor–effector proximity: nearest-neighbor distance distribution, used only after segmentation QC. Exhaustion-marker co-localization provides state context.	Relevant to CAR T-cell therapy, bispecific antibodies, checkpoint combinations, and immune escape.	T-cell abundance alone is insufficient; require functional state, location, and prospective response data.
Regulatory and myeloid suppressive niches	FOXP3 interpreted within a CD3+/CD4+ T-cell gate; PD-L1 assigned separately to tumor and myeloid lineages; myeloid interface quantified as boundary length or locked tile/bin area in which CD68+/CD163+/MRC1+ macrophages co-occur with tumor cells; myeloid–vessel proximity.	May identify suppressive hubs and rational contexts for macrophage-directed or immune-combination strategies.	Myeloid labels vary across platforms; require harmonized annotation and orthogonal protein-level validation.
Stromal and vascular compartmentalization	Stromal septa, fibroblast-rich regions, vessel density, vessel-to-tumor distance, and local cell-packing density.	May influence immune exclusion, antibody access, and local therapeutic delivery.	Interpret as lymphoma-specific tissue architecture, not automatically as a solid-tumor-like stromal barrier.
Candidate communication axes	PD-1/PD-L1, CD70/CD27, ICAM-1-related, chemokine-receptor, and antigen-presentation axes localized to defined niches.	Generates rational combination hypotheses and prioritizes mechanisms for testing.	Ligand-receptor inference is not proof of signaling; needs protein localization, perturbation, and non-overlapping coordinate-level validation.
Pathology-ready translational schema	Reduced FFPE panel: PAX5 or CD79a as a treatment-target-independent B-cell lineage backbone; CD20 retained as a target marker; CD3, CD4, CD8, CD68, PD-L1, FOXP3, and CD31, plus a therapy-matched target antigen such as CD19 or CD79b; optional MHC-I/MHC-II or B2M/HLA markers when antigen-presentation failure is tested.	Converts discovery findings into treatment-matched validation modules for CAR T-cell therapy, CD20×CD3 bispecific antibodies, ADC-containing therapy, and immune-combination trials.	Lock antibody clones, staining platform, ROI hierarchy, physical scale, segmentation fallback, and prespecified cutoffs before outcome testing; do not use as a clinical allocation algorithm without prospective validation.

ADC, antibody-drug conjugate; B2M, beta-2 microglobulin; CAR T-cell, chimeric antigen receptor T cell; DLBCL, diffuse large B-cell lymphoma; FFPE, formalin-fixed paraffin-embedded; HLA, human leukocyte antigen; MHC, major histocompatibility complex; PD-1, programmed cell death protein 1; PD-L1, programmed death-ligand 1; QC, quality control; ROI, region of interest; TAM, tumor-associated macrophage; Treg, regulatory T cell.

**Table 3:** Representative clinical trials and spatial-pharmacology validation hypotheses for immune-engaging DLBCL therapeutics.

Therapy class	Agent/trial and clinical signal	Key clinical context	Spatial-pharmacology hypothesis/resistance question	Pathology-ready spatial metric to validate
ADC-containing frontline	Polatuzumab vedotin; POLARIX, randomized double-blind phase 3; median follow-up 28.2 months: 2-year PFS 76.7% with pola-R-CHP vs 70.2% with R-CHOP (HR 0.73); no OS difference at primary analysis (5).	Previously untreated intermediate/high-risk DLBCL	CD79b heterogeneity; vascular delivery; candidate FcγR-mediated TAM processing or payload redistribution; cell-packing-dependent path tortuosity. These are validation hypotheses, not established causes of POLARIX failure.	CD79b density/heterogeneity by niche; CD68+/FcγR+ macrophage proximity to CD79b+ tumor; vessel-to-tumor distance; tile/bin target and payload-distribution proxies; temporal delta after therapy.
CAR T-cell therapy	Axicabtagene ciloleucel; ZUMA-7, randomized phase 3; median follow-up 24.9 months: median EFS 8.3 vs 2.0 months. Lisocabtagene maraleucel; TRANSFORM, open-label randomized phase 3 interim analysis; data cutoff 8 March 2021 and median follow-up 6.2 months: median EFS 10.1 vs 2.3 months versus standard care (29, 30).	Primary refractory or early-relapsed second-line large B-cell lymphoma	CD19 loss/modulation or trogocytosis-associated antigen transfer and density reduction; failed effector–target contact; macrophage-rich suppression; endogenous and infused T-cell exhaustion; broader immune-recognition defects, including impaired antigen presentation, interpreted as contextual rather than direct CAR-target escape.	Change in CD19 density; CAR-specific T-cell–tumor contact when detectable, with CD3+/CD8+ effector–tumor proximity used only as a surrogate; exhaustion-marker co-localization; myeloid-interface score; paired baseline/progression analysis at matched site and scale.
CD20×CD3 bispecific antibodies	Epcoritamab; phase I/II dose expansion; median follow-up 10.7 months: ORR 63.1%, CR 38.9%. Glofitamab; phase 2 fixed-duration cohort; median follow-up 12.6 months: ORR 52%, CR 39% in heavily pretreated LBCL/DLBCL cohorts (31, 32).	Relapsed/refractory disease after at least two prior lines in pivotal studies	Mismatch between local antibody-accessible fields and active T-cell migration; microvascular delivery heterogeneity; inadequate target density; Treg or macrophage-rich suppression. Diffusion failure remains unproven.	Tumor-T-cell proximity or field concordance; vessel density and vessel-to-tumor distance; CD20 patch size; CD3+/CD4+-gated FOXP3+ Treg enrichment; antibody-accessible area in explant/tracing studies.
Checkpoint blockade/combinations	Pembrolizumab; KEYNOTE-013 phase 1b (median follow-up 29.1 months) and KEYNOTE-170 phase 2 (median follow-up 12.5 months): clinically meaningful activity in relapsed/refractory PMBCL; transferability to conventional DLBCL is uncertain (33).	PMBCL and biomarker-selected large B-cell lymphoma contexts; investigational combinations in DLBCL	PD-1/PD-L1 abundance is insufficient without effector access, local macrophage context, antigen presentation, and tissue-site biology.	CD8 accessibility/exclusion in tumor ROIs confirmed by pathology and B-cell lineage markers; PD-L1+ tumor/myeloid interface; effector-to-PD-L1+ cell proximity; MHC status; site-stratified analysis.

Cross-trial caution: efficacy estimates are descriptive and should not be compared directly across rows because trial phase, eligibility, prior therapy, endpoint definitions, analysis timing, and follow-up differ. ADC, antibody-drug conjugate; CAR T-cell, chimeric antigen receptor T-cell; CR, complete response; DLBCL, diffuse large B-cell lymphoma; EFS, event-free survival; FcγR, Fc gamma receptor; HR, hazard ratio; LBCL, large B-cell lymphoma; MHC, major histocompatibility complex; ORR, overall response rate; OS, overall survival; PD-1, programmed cell death protein 1; PD-L1, programmed death-ligand 1; PFS, progression-free survival; PMBCL, primary mediastinal large B-cell lymphoma; pola-R-CHP, polatuzumab vedotin plus rituximab, cyclophosphamide, doxorubicin, and prednisone; R-CHOP, rituximab plus cyclophosphamide, doxorubicin, vincristine, and prednisone; ROI, region of interest; TAM, tumor-associated macrophage; Treg, regulatory T cell.

The review also aims to balance enthusiasm with evidence discipline. Spatial multi-omics can make previously invisible tissue relationships visible, but it can also create persuasive diagrams from indirect inference. A clinically useful synthesis must therefore distinguish what has been directly observed in tissue, what has been inferred from expression or proximity, and what has been functionally tested. This evidence-tier structure is used throughout the article to prevent promising spatial hypotheses from being presented as established mechanisms.

We use three terms deliberately. An ecosystem archetype refers to a biopsy- or sample-level microenvironmental class. A niche or cellular neighborhood refers to a local, coordinate-defined tissue unit within a sample. A communication axis refers to an inferred ligand-receptor or functional interaction that requires orthogonal validation. This distinction is central to the framework because a single archetype may contain several local niches, and an inferred axis should not be equated with a physical immune synapse or a causal pathway.

The main argument is that DLBCL resistance should be read through a genotype-to-niche-to-spatial-metric logic while preserving lymphoma-specific histology. Unlike many carcinomas, DLBCL often lacks a stable invasive front. Therefore, pathology-ready spatial metrics should prioritize tumor-rich regions of interest, local density fields, niche maps, and carefully quality-controlled cell-level distances rather than importing solid-tumor margin concepts. Detailed engineering rules, coordinate-level data standards, and cross-platform harmonization procedures are summarized in **Supplementary Tables S6–S8** so that the main text can focus on biological interpretation and immunotherapy-oriented translation. The conceptual genotype-to-niche-to-spatial-metric framework is summarized in **Figure 1**.

**Figure 1:**
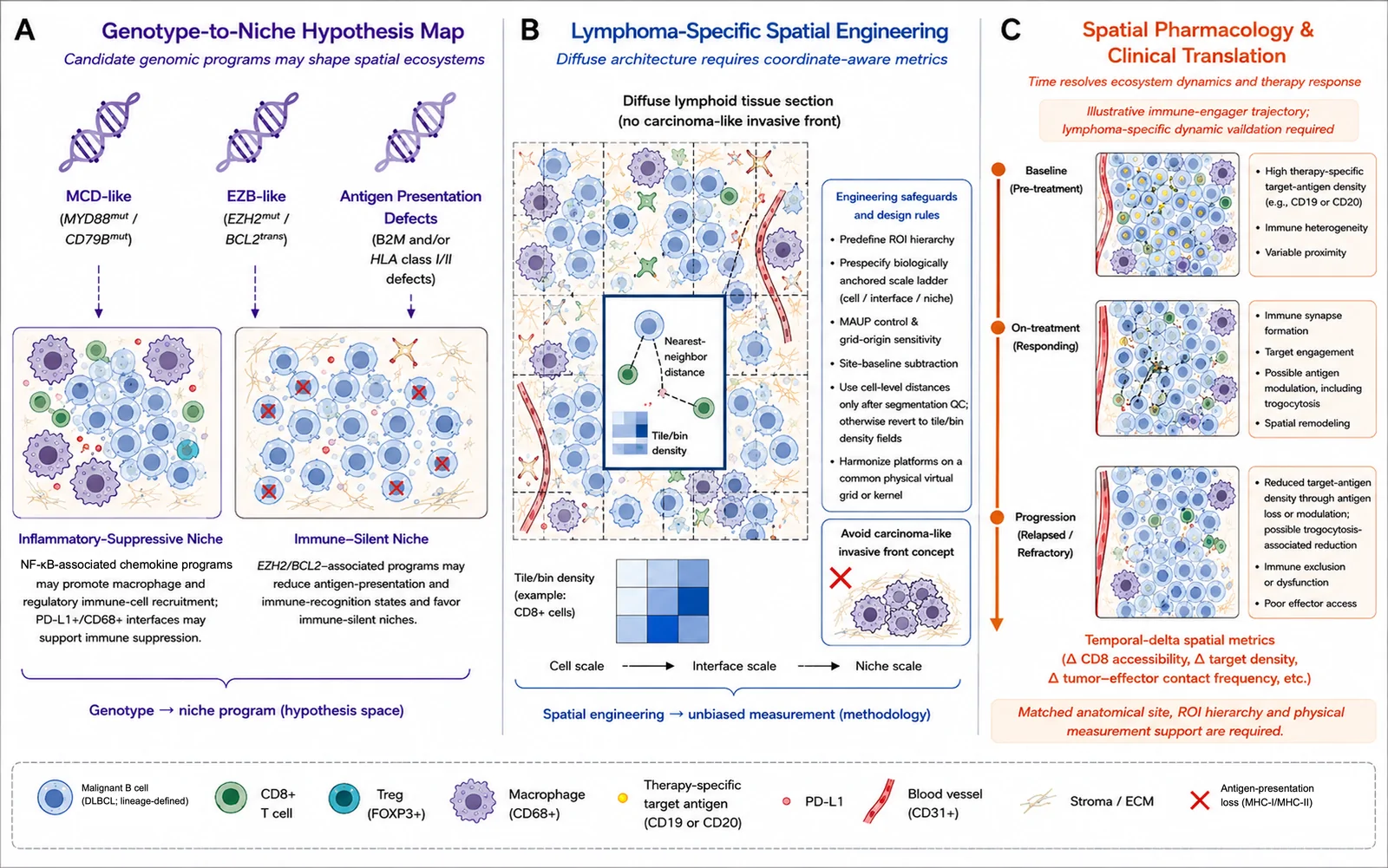
Translational rationale for spatial cancer-immunity profiling in DLBCL. **(A)** Genotype-to-niche hypothesis map. MCD-like and EZB-like genomic programs and defects in antigen presentation are linked to candidate inflammatory-suppressive or immune-silent spatial ecosystems. These relationships define a hypothesis space informed by convergent molecular and spatial evidence; they are not deterministic genotype-to-niche assignments or validated treatment-response predictors. **(B)** Lymphoma-specific spatial engineering. Because DLBCL commonly lacks a universal carcinoma-like invasive front, spatial analysis should use pathology-defined regions of interest, prespecified cell-, interface-, and niche-scale measurements, and locked tile/bin density fields. Segmentation quality control, scale sensitivity, site-aware baselines, and harmonization on a common physical support are methodological safeguards rather than biological findings. **(C)** Spatial pharmacology and clinical translation. The illustrative baseline, on-treatment, and progression sequence shows how therapy-matched target-antigen density, effector accessibility or contact, immune heterogeneity, and other spatial proxies could be evaluated longitudinally. Temporal-delta metrics require anatomically and technically comparable samples, a consistent ROI hierarchy, and identical physical measurement support. The depicted trajectory is conceptual, not a patient-derived reconstruction, an established treatment mechanism, or a clinically validated biomarker. ECM, extracellular matrix; MAUP, modifiable areal unit problem.

Accordingly, this Review prioritizes a cancer-immunology translation narrative over platform ranking. The central question is not which spatial platform is best, but which immune-resistance failure mode each platform can help test: loss of immune recognition, failed effector access, macrophage-dominated suppression, stromal or vascular compartmentalization, target-antigen heterogeneity, or treatment-associated spatial change. Technical terms such as coordinate-level validation, matrix-only reuse, virtual-grid support, and static transport proxies are retained only where they protect interpretation; detailed rules are placed in the **Supplementary Tables** so that the main article remains focused on immunological interpretation and therapy-matched validation.

## Literature selection and evidence synthesis approach

This is a structured narrative and mechanism-oriented Review rather than a systematic review or meta-analysis. PubMed/MEDLINE and Europe PMC were searched from database inception to 12 August 2026. The principal concepts were combined in two search blocks: (i) ‘diffuse large B-cell lymphoma’, ‘DLBCL’, or ‘large B-cell lymphoma’ with ‘single-cell’, ‘single-nucleus’, ‘spatial transcriptomics’, ‘spatial proteomics’, ‘digital spatial profiling’, ‘imaging mass cytometry’, ‘multiplex immunofluorescence’, ‘tumor microenvironment’, ‘immune’, ‘macrophage’, ‘T cell’, or ‘resistance’; and (ii) the same disease terms with ‘CAR T’, ‘bispecific antibody’, ‘antibody-drug conjugate’, ‘polatuzumab’, or ‘checkpoint’, together with ‘microenvironment’, ‘spatial’, ‘resistance’, or ‘biomarker’. Reference lists of eligible articles and relevant reviews were also screened for additional primary studies. Search reporting and critical appraisal were guided by the Scale for the Assessment of Narrative Review Articles (SANRA) recommendations for narrative reviews (34).

Eligible reports were English-language, peer-reviewed primary studies or pivotal clinical-trial reports that addressed DLBCL or broader LBCL ecosystems, spatial or single-cell organization, immune escape, antigen presentation, treatment resistance, therapy-matched biomarkers, or functional validation models. Reviews were used to identify context and additional primary sources but were not counted as core evidence. Conference abstracts without sufficient methodological detail, non-peer-reviewed reports, studies unrelated to LBCL, and purely computational analyses without a relevant primary dataset were not treated as core evidence. Because this was a narrative synthesis, study selection and evidence charting were not performed as a preregistered, duplicate-screened systematic-review workflow; no pooled effect estimate or quantitative risk-of-bias score was calculated.

Studies were interpreted according to evidence support rather than counted as equivalent observations. Coordinate-preserving spatial studies, independent orthogonal or protein-level validation, outcome or treatment-response associations, and genuinely predictive evidence were recorded separately. **Supplementary Table S1** charts 16 core high-resolution studies that met the scope criteria, and Main **Table 1** presents nine deliberately selected representatives—including Ferrant et al.—chosen to span foundational ecosystem modeling, descriptive single-cell atlases, coordinate-preserving imaging or transcriptomic studies, multimodal DLBCL niche mapping, LBCL-wide archetype profiling, and vascular–myeloid niche validation. The studies selected for **Table 1** represent complementary evidence types rather than a numerical evidence count. Additional supporting summaries are provided in **Supplementary Tables S2**–**S5**. Associative, inferred, or context-dependent findings are explicitly labeled as candidate hypotheses or validation priorities. **Supplementary Figure 1** provides a visual summary of this evidence structure and its non-exclusive streams.

## Malignant B-cell states and tumor-intrinsic resistance programs

High-resolution studies consistently show that malignant B cells in DLBCL are not transcriptionally uniform. EcoTyper-based and single-cell studies identified recurrent malignant B-cell states, ecosystem configurations, and immune compartments across patients (10, 11). Subsequent studies connected malignant programs to survival, metabolic adaptation, autophagy-related signatures, antigen-presentation remodeling, and adverse immune states (13, 16, 21, 22, 24). These data support a view in which tumor-intrinsic programs help organize local immune ecology rather than acting as isolated cell-autonomous traits.

The relative evidentiary weight of these studies is important. Multimodal spatial characterization of DLBCL tissues, large B-cell lymphoma microenvironment archetype profiling, and myeloid-endothelial co-localization studies provide the strongest recent support for the niche-centered framework because they link high-dimensional cell states to tissue organization across relatively informative cohorts (22, 23, 25). Integrative single-cell or bulk analyses, including ABC-type DLBCL studies that nominate CD70/CD27, ICAM-1-related, or TYROBP-associated programs, are valuable but should be read as hypothesis-generating support unless accompanied by coordinate-preserving spatial validation and outcome-linked tissue context (24).

The genotype-to-niche concept is useful but should remain hypothesis-generating. MCD-like biology, characterized by MYD88/CD79B-related chronic B-cell receptor, Toll-like receptor, and NF-κB signaling, may favor inflammatory, interferon-response, checkpoint, chemokine, and macrophage-rich programs. EZB-like biology, linked to EZH2/BCL2-associated pathways, may favor immune-silent or proximity-limited states through epigenetic maintenance, apoptosis resistance, and reduced immune recognition. Defects affecting B2M or MHC-I antigen presentation may impair direct recognition by CD8+ T cells even when these cells are numerically present, whereas MHC-II defects may impair antigen recognition by CD4+ T cells (8, 9, 35, 36).

A practical example is antigen presentation. A tumor with B2M, HLA, or MHC pathway disruption may still contain CD8+ T cells, but those T cells may be unable to recognize antigen-competent malignant B cells productively. The relevant spatial readout is therefore not simple CD8+ abundance; it is the distribution of CD8+ cells relative to malignant B-cell regions with preserved or lost antigen-presentation markers. Similarly, an inflammatory MCD-like tumor should not automatically be labeled immune active. It may instead produce a macrophage-rich or PD-L1-enriched niche that recruits immune cells but diverts them into suppressive or exhausted states.

These relationships should not be interpreted as deterministic rules. MCD-like disease is enriched in immune-privileged sites, and nodal, primary central nervous system (CNS), testicular, Epstein-Barr virus (EBV)-positive, and relapsed lesions can differ in resident macrophage biology, vascular access, stromal architecture, and baseline T-cell entry (35). A genotype-associated niche may therefore reflect organ anatomy as much as tumor-driven niche construction. The preferred validation strategy is within-site comparison, followed by genotype-by-site interaction testing and, when necessary, rare-site fallback approaches as detailed in **Supplementary Table S7**.

This site-aware framing is particularly important for primary CNS and testicular lymphoma. In these settings, immune privilege, resident macrophage or microglial programs, vascular specialization, and tissue-specific stromal support may shape the baseline ecosystem before tumor genotype is considered. A spatial metric that appears tumor-genotype-associated in a pooled cohort may disappear when nodal and extranodal cases are separated. For this reason, anatomical site is treated as part of the biological model rather than a nuisance variable to be adjusted away after discovery.

Metabolism provides one mechanistic bridge between malignant B-cell state and immune context. High-glycolysis malignant B-cell programs have been associated with tumor-associated macrophage clusters and exhausted immune features in DLBCL, suggesting a testable metabolic-immune niche (16). More broadly, lactate-rich and nutrient-competitive microenvironments can suppress effector T-cell metabolism and support myeloid remodeling, although direct causal evidence in DLBCL remains limited (37, 38).

These examples show why malignant B-cell biology and microenvironment biology should be interpreted together. A purely genomic classifier can identify likely upstream programs, but it cannot determine whether immune cells are excluded, suppressed, or functionally engaged in a particular tissue region. Conversely, a purely spatial immune score can describe a local pattern but may miss the tumor-cell program that generated or maintained that pattern. The proposed genotype-to-niche-to-metric model is intended to link these layers without converting an association into a deterministic clinical rule.

## T-cell dysfunction and regulatory immune remodeling

T-cell abundance alone is insufficient to define immune competence in DLBCL. Spatial immune profiling, imaging mass cytometry, high-parameter imaging, and multimodal spatial profiling have shown that T cells can be unevenly distributed across tumor-rich, immune-rich, myeloid-rich, and stromal compartments (12, 14, 17, 22). In some niches, immune cells may be close enough to malignant cells to support engagement; in others, they may be excluded, exhausted, or separated by myeloid or stromal structures.

Regulatory immune remodeling further complicates interpretation. FOXP3+ regulatory T cells, exhausted CD8+ T cells, and dysfunctional effector phenotypes may occupy different regions of the same biopsy and may interact with macrophage-rich or tumor-rich compartments in distinct ways. A sample-level immune score can therefore obscure whether a case is immune-active, immune-excluded, immune-suppressed, or simply spatially heterogeneous. Future studies should report T-cell state, location, and local cellular partners together rather than treating them as separable variables.

Single-cell and spatially informed studies further indicate that exhausted CD8+ T-cell states, regulatory T-cell features, and dysfunctional effector programs can carry prognostic or therapeutic relevance (39, 40). T cells within tumor-B-cell-rich niches may express dysfunction-associated markers such as ENTPD1, TIGIT, and PDCD1, whereas T-cell-enriched regions can show distinct recruitment and activation patterns (22). These observations are clinically important because CAR T-cell therapy and bispecific antibodies require functional effector cells, sufficient target density, and productive tumor-effector contact.

The translational implication is that future immune biomarkers should measure both state and position. A CD8+ T-cell score is more informative when it specifies whether CD8+ cells are inside pathologist-confirmed tumor-rich regions supported by B-cell lineage markers, restricted to adjacent compartments, enriched near PD-L1+ macrophages, or associated with exhaustion markers. This principle underlies the pathology-ready CD20/CD3/CD8 and CD20/CD68/PD-L1 metrics summarized in **Table 2** and **Supplementary Table S8**.

This is especially relevant for treatment response. CAR T cells and bispecific antibodies are not passive biomarkers; they are living or immune-engaging therapies whose efficacy depends on migration, persistence, contact, activation, and resistance to local suppression. A pretreatment biopsy that contains many CD8+ cells but little tumor-effector proximity may not represent the same biological state as a biopsy with fewer but better-positioned effector cells. Longitudinal samples are needed to determine whether treatment restores contact, deepens exclusion, or selects antigen-low resistant neighborhoods.

## Myeloid, stromal, and vascular niches

Myeloid remodeling is a central component of the DLBCL ecosystem. Spatial transcriptomic and imaging studies have identified macrophage heterogeneity, macrophage-derived spatial signatures, suppressive vascular-myeloid niches, and fibroblast-macrophage neighborhoods with prognostic or biological relevance (15, 20, 22, 25). These findings argue against treating macrophages as a homogeneous background population. Instead, interferon-associated TAMs, FN1+ macrophage-associated regions, PD-L1+ macrophage interfaces, and endothelial-associated myeloid niches may represent distinct contexts of immune suppression or remodeling.

The myeloid compartment is also a major reason why spatial analysis may outperform bulk immune abundance. Macrophages can localize near tumor cells, blood vessels, fibroblast-rich regions, or residual lymphoid architecture, and each location may imply a different biological role. A CD68-high tumor could reflect phagocytic activity, antigen-presentation remodeling, interferon-associated inflammation, suppressive PD-L1-rich interfaces, or vascular-associated myeloid niches. Without spatial localization and marker context, these possibilities are difficult to distinguish.

Stromal and vascular compartments add another layer of spatial regulation. Fibroblast-rich regions, stromal septa, vessel-adjacent areas, and local cellular packing may influence immune access, antibody exposure, and myeloid localization (20, 22, 25). However, DLBCL-specific tissue architecture should not be collapsed into a generic solid-tumor barrier model. Lymph-node, CNS, testicular, and other extranodal compartments differ in baseline anatomy and immune privilege, so transport and access hypotheses must be tested within relevant tissue contexts.

For clinical translation, the safest language is therefore “compartmentalization” rather than “barrier” unless dynamic evidence is available. Static histology can show that vessels, stromal septa, macrophages, and tumor-rich sheets are arranged in ways that may influence access, but it cannot directly measure antibody diffusion, payload release, receptor-mediated consumption, convection, or immune-cell chemotaxis. Those mechanisms require paired tissue, drug or payload tracing, functional models, or carefully designed proxy validation.

Large B-cell lymphoma microenvironment archetype profiling complements niche-level mapping by defining sample-level ecosystem classes across biopsies (23, 41). Archetypes can stratify broad tumor microenvironments, whereas local niche analysis explains how immune-active, macrophage-rich, stromal-dominant, or tumor-rich regions coexist within a single biopsy. The two concepts are therefore complementary but not interchangeable. This archetype-to-niche scale distinction and representative spatial states are illustrated in **Figure 2**.

**Figure 2:**
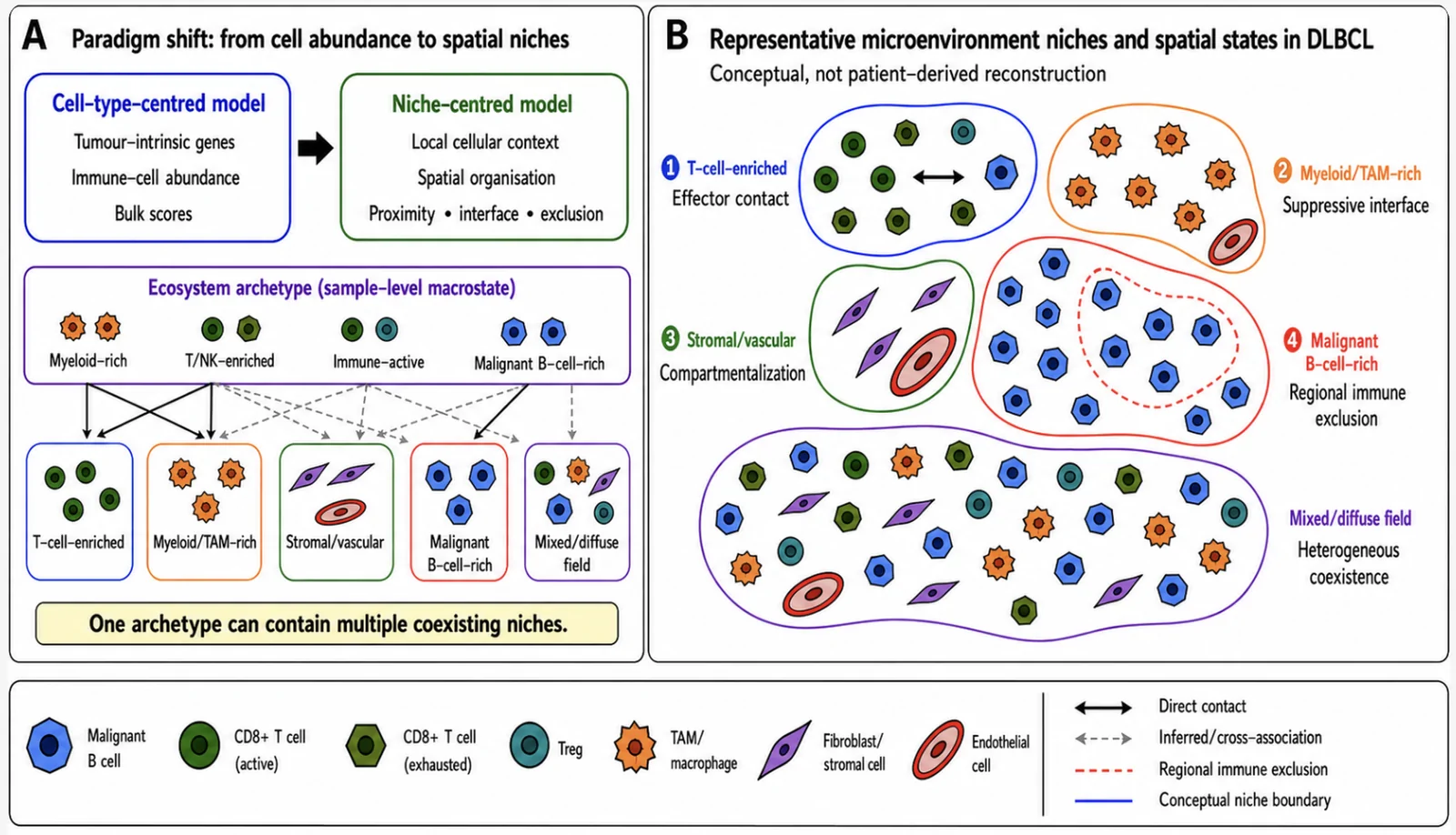
Niche-centered framework of immune organization and spatial states in DLBCL. **(A)** Comparison of the conventional cell-type-centered model with a niche-centered framework. Ecosystem archetype denotes a sample-level or biopsy-level macrostate, whereas niche denotes a coordinate-defined local spatial unit. Solid arrows indicate illustrative primary associations and gray dashed arrows indicate inferred or cross-associations; these relationships are non-deterministic, and multiple niches may coexist within one archetype. **(B)** Conceptual representation of T-cell-enriched, myeloid/TAM-rich, stromal/vascular, and malignant B-cell-rich niches, together with a mixed/diffuse spatial field. Solid colored outlines denote conceptual niche boundaries, the black bidirectional arrow denotes illustrative effector–tumor contact, and the red dashed boundary denotes regional immune exclusion. The patterns are a mechanism-generating synthesis rather than patient-derived reconstruction, experimentally validated receptor engagement, or a proven clinical-classification algorithm.

This separation helps align the framework with recent higher-level evidence. Archetype studies can define broad immune-rich, stromal-rich, macrophage-rich, or tumor-dominant ecosystems across large cohorts, while spatial niche studies can identify the local structures that make those archetypes clinically meaningful. For example, a macrophage-rich archetype may include suppressive macrophage-tumor interfaces, vascular-associated myeloid hubs, or fibroblast-macrophage neighborhoods. These are not interchangeable mechanisms, and their therapeutic implications may differ.

## Spatial communication axes and treatment implications

Spatial and single-cell studies nominate several recurrent communication axes with therapeutic relevance, including PD-1/PD-L1, CD70/CD27, ICAM-1-related interactions, chemokine-receptor programs, and antigen-presentation pathways (12, 19, 22, 24). In ABC-type DLBCL, integrative analysis has suggested CD70-CD27 signaling associated with regulatory or exhausted T-cell programs and ICAM-1-related interactions in more favorable contexts (24). Because this evidence is largely computational and study-specific, these axes should be considered emerging candidates rather than independently validated mechanisms.

Findings reproduced in treatment-annotated, coordinate-preserving, protein-supported, and non-overlapping cohorts should be treated as stronger evidence than axes recovered from a single computational pipeline or from matrix-only reanalysis. This does not diminish emerging studies; rather, it prevents useful hypotheses from being overstated. In the present framework, CD70/CD27 and ICAM-1-related signals are positioned as promising candidates for mechanistic testing, not as established DLBCL resistance pathways.

A niche-centered resistance framework separates three evidence levels: observed cell states and tissue neighborhoods; computationally inferred communication or co-localization programs; and experimentally validated functional interactions. Only the last level justifies causal language. This distinction is especially important when similar ligand-receptor axes recur across studies that share patients, reference atlases, public matrices, or communication databases.

The clinical value of spatial biology is therefore not that it immediately selects treatment, but that it defines what must be measured in treatment-matched tissue. For immune therapies, relevant questions include whether effectors enter pathologist-confirmed tumor-rich regions supported by B-cell lineage markers, whether target antigen remains dense and accessible, whether macrophage-rich niches suppress local function, and whether therapy remodels these relationships over time. These principles are summarized in **Figure 3** and **Tables 2**, **3**.

**Figure 3:**
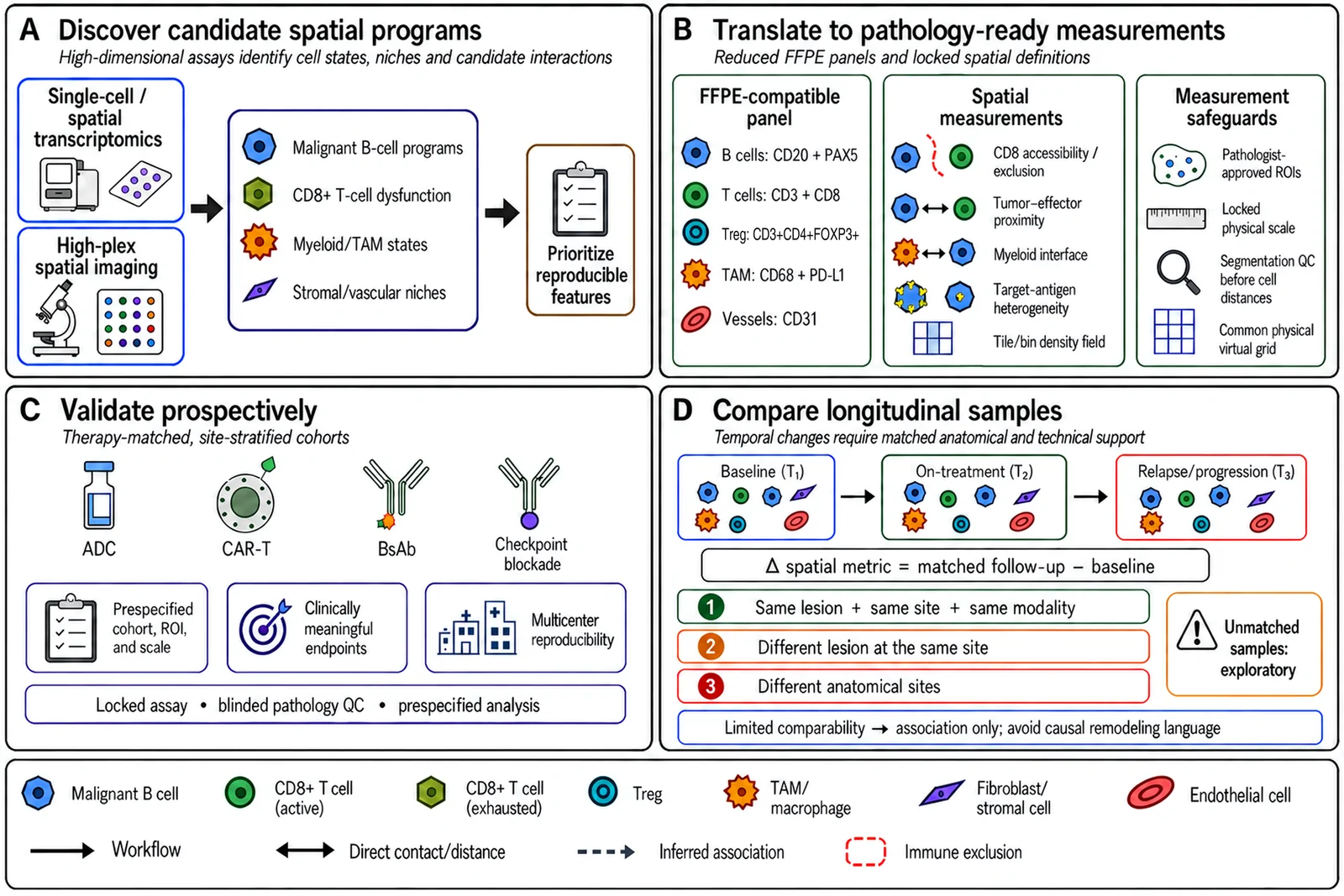
Pathology-aware spatial translation and longitudinal validation of immune-ecosystem biomarkers in DLBCL. **(A)** High-dimensional single-cell or spatial transcriptomics and high-plex spatial imaging identify candidate malignant B-cell programs, CD8+ T-cell dysfunction, myeloid/TAM states, and stromal or vascular niches. These features are prioritized for translation and should not be interpreted as validated biomarkers. **(B)** Candidate features are reduced to FFPE-compatible marker panels and locked spatial measurements. For the Treg module, include CD4 and interpret FOXP3 within a CD3+/CD4+ T-cell gate. PAX5 or CD79a should be retained as a treatment-target-independent B-cell lineage anchor, whereas CD20 is retained as the therapy-target readout, particularly after anti-CD20 therapy. CD8 accessibility or exclusion describes the prespecified distribution of CD8+ cells within or relative to tumor-rich ROIs or tile/bin fields; tumor–effector proximity represents a segmentation-quality-controlled distance or contact metric; the myeloid-interface metric captures enrichment or contact of CD68+/PD-L1+ macrophages at tumor–immune boundaries; target-antigen heterogeneity describes spatial variation in therapy-matched antigens such as CD19, CD20, or CD79b; and tile/bin density fields aggregate cell or marker density over locked physical units. Pathologist-approved ROIs, physical scale, segmentation quality-control requirements, and the cross-platform virtual grid should be prespecified. **(C)** Candidate biomarkers should be evaluated in therapy-matched and anatomically stratified prospective cohorts using locked assays, clinically meaningful endpoints, blinded pathology quality control, prespecified analyses, and multicenter reproducibility testing. **(D)** Longitudinal comparisons should prioritize samples from the same lesion, anatomical site, and biopsy modality, followed by different lesions from the same site and, lastly, samples from different anatomical sites. Temporal-delta metrics are defined as the matched follow-up measurement minus baseline on the same physical support. Unmatched or poorly comparable samples should be considered exploratory; such comparisons support association rather than causal claims of treatment-induced spatial remodeling. Solid black arrows indicate workflow progression, bidirectional black arrows indicate direct contact or distance, gray dashed arrows indicate inferred associations, and red dashed boundaries indicate immune exclusion. The workflow is a proposed validation framework rather than a proven clinical algorithm. BsAb, bispecific antibody.

## Minimal pathology-ready validation schema

A practical translational schema should begin with a reduced FFPE-compatible panel rather than a maximal discovery assay. A core lymphoma spatial panel could include PAX5 or CD79a as a treatment-target-independent B-cell lineage anchor, with CD20 retained as a therapy-target readout; effector T-cell markers (CD3 and CD8); macrophage and suppressive-interface markers (CD68 with lineage-resolved PD-L1); FOXP3 interpreted within a CD3+/CD4+ T-cell gate; vascular support (CD31); and a therapy-matched target antigen selected according to treatment context, such as CD19 for CAR T-cell therapy, CD20 for CD20×CD3 bispecific antibodies, or CD79b for polatuzumab-containing regimens. Optional MHC-I/MHC-II or B2M/HLA markers can be added when antigen-presentation failure is the primary hypothesis.

This panel should then be analyzed through treatment-matched spatial modules. For CAR T-cell therapy, the priority readouts are CD19 target-antigen density, endogenous or CAR-specific T-cell localization when detectable, tumor-effector proximity, and a myeloid-interface score. For CD20×CD3 bispecific antibodies, the priority readouts are CD20-rich tumor fields, CD3/CD8 effector proximity, FOXP3 or macrophage-associated suppression, and vessel-supported access. For ADC-containing regimens, the priority readouts are CD79b spatial heterogeneity, vessel-to-tumor distance, tumor-cell packing density, and macrophage proximity. These modules should be interpreted as trial-ready validation schemas rather than clinical allocation rules until prospective treatment-matched cohorts demonstrate incremental predictive value beyond standard clinicogenomic variables. The corresponding candidate mechanisms, measurable proxies, and evidence caveats are summarized in **Supplementary Figure 2**.

## Treatment-context interpretation

For frontline immunochemotherapy and ADC-containing therapy, clinical trial results establish efficacy but not spatial mechanism. In POLARIX, pola-R-CHP improved 2-year progression-free survival compared with R-CHOP, while overall survival was not different at the primary analysis (5). Spatial studies can generate hypotheses about why individual tumors fail these regimens, including malignant-cell metabolic adaptation, antigen-presentation remodeling, CD79b heterogeneity, vascular delivery, cellular packing, and candidate macrophage-mediated ADC processing. These remain validation hypotheses, not proven causes of pola-R-CHP failure (5, 6, 42, 43).

The same caution applies to ADC spatial pharmacology. Polatuzumab vedotin creates a therapeutic context in which CD79b expression, internalization, lysosomal processing, bystander exposure, and local macrophage interactions may matter, but routine baseline sections cannot determine which of these mechanisms dominates in a patient. Antibody size, tissue penetration, stromal context, and extracellular matrix composition can influence local distribution, so baseline image-derived features should be treated as transport proxies rather than direct measurements of antibody diffusion or payload movement (44, 45). A pathology-ready validation plan should therefore begin with measurable features such as CD79b patch heterogeneity, cellular packing assessed with DAPI and B-cell lineage markers within pathologist-confirmed tumor regions, vessel-to-tumor distance, and CD68+/FcγR+ macrophage proximity, and then test whether these proxies correlate with drug localization or clinical response.

For CAR T-cell therapy, randomized second-line trials demonstrated clinical superiority over standard care in primary refractory or early-relapsed large B-cell lymphoma (29, 30). Spatial resistance hypotheses include CD19 loss or modulation, trogocytosis-associated antigen transfer, failed effector-target contact, exhausted T-cell neighborhoods, macrophage-rich suppression, and broader immune-recognition defects (46, 47). These mechanisms should be tested with paired antigen-density, effector-localization, and functional-state readouts rather than inferred from baseline immune abundance alone.

The CAR T-cell setting also illustrates why spatial biomarkers should be therapy-matched. Endogenous CD8+ T-cell proximity is not identical to infused CAR T-cell localization, and CD19 antigen loss is not identical to broader antigen-presentation failure. Nevertheless, pretreatment microenvironmental context can influence expansion, persistence, trafficking, and local function. Spatial assays should therefore distinguish target-antigen density, CAR-specific localization when available, endogenous effector surrogates, macrophage-rich suppression, and temporal changes at relapse.

For CD20×CD3 bispecific antibodies, pivotal studies of epcoritamab and glofitamab reported meaningful responses in heavily pretreated large B-cell lymphoma (31, 32). Target expression is necessary but unlikely to be sufficient. Local T-cell fitness, tumor-effector proximity, regulatory T-cell enrichment, macrophage-mediated suppression, vascular access, and antibody-accessible fields may determine whether engagement becomes cytotoxicity. A mismatch between passive antibody distribution and active immune-cell movement is plausible, but it should be described as a site-specific spatial-pharmacology hypothesis until directly measured.

For bispecific antibodies, the key readout is not merely whether CD20 and CD3 are present somewhere in the section, but whether a local tissue context permits effective engagement. A tumor-rich niche with high CD20 density and nearby functional T cells may be biologically different from a tumor-rich niche separated from T cells by stromal or myeloid compartments. Future trials should prioritize paired baseline and early on-treatment biopsies because synapse formation, antigen modulation, and immune exhaustion may evolve quickly after exposure to T-cell-engaging therapy.

For checkpoint and myeloid-directed combinations, spatial context may help identify rational trial settings. Pembrolizumab has activity in relapsed or refractory primary mediastinal large B-cell lymphoma, but that result should not be generalized to unselected DLBCL (33). PD-1/PD-L1 expression, macrophage abundance, and myeloid programs are most interpretable when localized to the neighborhoods in which the relevant effector, suppressive, stromal, or tumor cells actually coexist.

Across treatment classes, the most important unanswered question is temporal directionality. A resistant niche may exist before therapy, emerge under selective pressure, or appear because the accessible lesion sampled at relapse is not the same biological compartment sampled at diagnosis. Static baseline sections can nominate candidate risk states, but paired biopsies are needed to determine whether therapy reduces target antigen, increases macrophage-dominated suppression, restores effector access, or selects spatially protected clones. This is why temporal-delta metrics are emphasized as a validation priority rather than a routine clinical tool. Key evidence boundaries for interpreting these treatment-specific hypotheses are summarized in **Box 1**.

Box 1 **Evidence boundaries for interpretation and clinical translation.**

- Frontline and ADC-containing therapy: spatial profiling may refine interpretation of R-CHOP or pola-R-CHP failure, but ADC access, payload redistribution, and macrophage-processing models should remain explicitly labeled as lymphoma-specific hypotheses.
- CAR T-cell therapy: resistance may involve target-antigen loss or modulation, reduced productive contact, exhausted effector states, macrophage-rich suppression, and immune-anatomical compartmentalization.
- Bispecific antibodies: response should be studied through target density, local effector fitness, tumor-T-cell proximity, regulatory or myeloid suppression, and antibody-accessible tissue fields.
- Static tissue metrics: FFPE images provide proxy indices for packing, vessel distance, target patches, and macrophage proximity; they do not directly measure diffusion, convection, receptor consumption, or chemotaxis.
- Clinical use: current spatial models are best viewed as trial-design tools and candidate biomarker frameworks, not as established clinical allocation rules.

## Discussion

Single-cell and spatial multi-omics are changing the interpretation of lymphoma immunotherapy resistance by shifting attention from isolated cell types to spatially organized cancer-immune niches. A cell-type-centered model asks whether a tumor contains malignant B cells, CD8+ T cells, macrophages, or stromal cells. A niche-centered model asks where these cells are located, what functional states they occupy, and whether their arrangement permits immune recognition, local suppression, drug access, or treatment-associated spatial change.

This distinction matters clinically. A tumor with abundant T cells may remain immune resistant if those cells are exhausted, spatially excluded from pathologist-confirmed tumor regions supported by B-cell lineage markers, suppressed by adjacent macrophages, or trapped in non-tumor compartments. Conversely, a tumor with moderate immune infiltration may be more vulnerable if effector cells retain function and localize near antigen-competent malignant cells. Spatial context may therefore convert immune biomarkers from abundance measures into architecture-informed readouts.

The evidence base also refines the role of tumor-intrinsic programs. Malignant B-cell metabolism, antigen presentation, checkpoint-related signaling, chemokine secretion, and epigenetic state are likely to shape the local immune environment. However, most current data are cross-sectional. The field should avoid declaring causal genotype-driven niche construction unless the association is reproduced within anatomical sites, supported by functional perturbation, and linked to treatment response.

For therapeutic development, the immediate value of this literature is rational hypothesis generation. CAR T-cell and bispecific-antibody resistance may require combinations that restore effector fitness, improve tumor-effector contact, or reduce regulatory and macrophage-mediated suppression. ADC-containing therapy may require spatial readouts of target heterogeneity, vascular access, cellular packing, macrophage proximity, and payload distribution. Checkpoint or myeloid-directed combinations should be tested in niches where the relevant suppressive and effector components coexist.

## Functional validation systems and companion-diagnostic development

Most DLBCL spatial studies remain observational, so causal validation requires model systems that preserve multicellular context. Conventional cell lines are tractable but lack human immune, stromal, and tissue organization; patient-derived xenografts preserve malignant genetics but often replace key microenvironment components. Patient-derived lymphoma spheroids, primary 3D co-cultures, tissue explants, and perfused microfluidic or organotypic systems provide intermediate platforms for testing antibody exposure, macrophage uptake, antigen transfer, and effector migration (48, 49).

Model selection should match the claim being tested. A simple co-culture may be adequate for receptor engagement or cytotoxicity, but it cannot validate vascular access or tissue-scale compartmentalization. A spheroid or organoid-like culture can test cell packing and multicellular contact but may not reproduce organ-specific vasculature or immune privilege. Tissue explants and microfluidic systems are better suited for time-resolved antibody, ADC, payload, macrophage-uptake, or effector-migration experiments, but their conclusions still need benchmarking against patient tissue.

Translation should proceed as a staged biomarker program rather than immediate companion-diagnostic deployment. Candidate assays could include FFPE-compatible CD20/CD3/CD8 accessibility scores or CD20/CD68/PD-L1 myeloid-interface scores, with optional FOXP3, exhaustion, stromal, vascular, or target-antigen markers selected by treatment context. Analytical validation should precede clinical claims and include antibody performance, inter-run and inter-laboratory reproducibility, scanner transfer, pathologist ROI agreement, segmentation fallback, and locked physical scale. Until predictive value beyond clinicogenomic standards is shown prospectively, these assays should be called candidate spatial stratification tools rather than regulatory-grade companion diagnostics.

For operational use, effector-cell accessibility is assessed using CD3+/CD8+ cell density within a pathologist-approved tumor ROI and the fraction of lineage-defined tumor cells with an effector cell inside a prespecified physical radius, provided that the required cell identification and segmentation pass quality control. Tumor-effector proximity is summarized by the nearest-neighbor distance distribution under the same quality-control requirement. In regions that fail this requirement, measurements that depend on the failed segmentation should not be reported. Separately validated ROI- or tile/bin-level marker-density readouts may be used where they do not rely on the failed segmentation. A myeloid interface is the boundary length or locked tile/bin area in which lineage-confirmed macrophages co-occur with tumor cells; PD-L1 must be assigned separately to tumor, myeloid, or other lineages. Target-antigen heterogeneity is the within-tumor-compartment distribution of per-cell or patch-level intensity, including the antigen-low fraction. Temporal delta is the follow-up minus baseline value calculated with unchanged anatomical-site tier, biopsy modality, ROI hierarchy, physical support, and normalization. FOXP3 should be interpreted within a T-cell lineage gate, such as CD3+/CD4+, and after anti-CD20 therapy the tumor compartment should be anchored by a treatment-target-independent B-cell marker such as PAX5 or CD79a rather than CD20 alone.

The proposed assays should also be evaluated against standard clinical and genomic variables. A spatial score that is merely correlated with cell of origin, International Prognostic Index, tumor burden, anatomical site, or known genomic subtype may still be biologically interesting, but it is not yet a useful biomarker. The translational goal is incremental value: improved risk stratification, prediction of treatment interaction, or selection of rational combination therapy beyond existing clinicopathologic and genomic tools.

## Critical appraisal of evidence and platforms

Evidence independence must be interpreted more strictly in this field. A communication axis is not independently validated merely because it appears in multiple analyses if those analyses reuse overlapping patients, processed matrices, reference atlases, cell-state labels, or ligand-receptor databases. Reanalysis of expression matrices without coordinates can be useful for sample-level ecosystem or pathway hypotheses, but it cannot reproduce local geometry, exclusion, proximity, interface, synapse, or temporal-remodeling claims (50, 51). For this reason, reusable spatial evidence should preserve links among expression matrices, coordinate files, tissue images, masks, scale factors, anatomical-site labels, clinical annotations, and analysis code; tumor-atlas and spatial-data resources provide useful models for this level of metadata and accessibility (52–56).

Spatial findings should be interpreted according to the physical scale and unit of measurement, anatomical context, and level of evidence; computational inference should not be equated with causal biology.

Platform resolution also defines what can be claimed. Dissociated single-cell RNA sequencing provides cellular transcriptional resolution but loses spatial context. Spot-based spatial transcriptomics offers regional whole-transcriptome data but may mix multiple cells within a spot. Emerging high-resolution spatial transcriptomic platforms, including DNA nanoball-patterned array-based approaches, have expanded the physical resolution of spatial mapping, although interpretation still depends on measurement support and tissue context (56–59). Cell-resolved *in situ* RNA platforms and high-plex protein imaging, including platforms such as Xenium, CosMx, PhenoCycler-Fusion, and imaging mass cytometry, are better suited for neighborhood and proximity analysis, but they remain limited by targeted panels, segmentation quality, fixation, and registration (56, 60–65). Cross-platform validation should therefore target a shared physical support, such as a density field or virtual grid, rather than claiming that a spot-based dataset confirms cell-cell contact. A platform-aware comparison is provided in **Table 4**.

**Table 4:** Platform-aware comparison of spatial multi-omics technologies for DLBCL ecosystem profiling.

Platform	Modality	Native spatial support/breadth	Best DLBCL use case	Key limitation and interpretation rule
Visium (10x)	Sequencing-based spatial RNA	55 µm multicellular spots; whole-transcriptome or probe-based transcriptome depending on the assay (57).	Unbiased regional discovery across tumor-rich, immune-rich, stromal, and vascular compartments.	Spot mixing prevents direct cell-contact or nearest-neighbor claims. Use deconvolution/field reconstruction and common-support virtual binning for cross-platform comparison.
Visium HD (10x; probe-based assay)	Sequencing-based spatial RNA	Continuous 2 × 2 µm capture squares; commonly visualized/aggregated at 8 or 16 µm; whole-transcriptome probe panel (58).	High-resolution mapping of dense lymphoid sheets and regional expression gradients while retaining discovery breadth.	Single-cell-scale is not automatically cell-resolved. Bin aggregation, segmentation, capture efficiency, and cell mixing require sensitivity analyses.
Xenium Prime 5K (10x)	Imaging-based *in situ* RNA with morphology/protein options	Targeted up to 5,000 genes plus customization; transcript coordinates and cell segmentation at subcellular localization (60).	Cell-resolved neighborhood, proximity, target-antigen, and candidate synapse-support analysis in FFPE tissue.	Panel-dependent; segmentation errors in dense DLBCL can bias contact metrics. Validate with tile/bin density and morphology QC.
CosMx SMI (Bruker; 6K Discovery assay)	Imaging-based spatial RNA/protein	Targeted ~6,000 RNA genes with optional custom targets and protein-assisted segmentation; single-cell/subcellular localization (61).	Cell-state and ligand-receptor localization across large FFPE tissue regions with targeted high plex.	Targeted panel may omit unanticipated genes; segmentation and transcript-assignment uncertainty must be reported.
Imaging mass cytometry (IMC)	Antibody-based spatial proteomics	Subcellular laser-ablation imaging; typically tens of protein markers (28, 65).	Protein-level immune-state, checkpoint, phospho-signaling, and cell-neighborhood validation.	Antibody panel, fixation, ablation area, normalization, and segmentation constrain discovery and throughput.
PhenoCycler/CODEX	Cyclic antibody-based spatial proteomics	Single-cell whole-slide imaging; panels can exceed 100 biomarkers in current implementations (64).	Large-area multiplex phenotyping, rare niche detection, and reduced-panel validation.	Antibody validation, cyclic imaging artifacts, autofluorescence, bleed-through, and cell segmentation require explicit QC.
Stereo-seq	Sequencing-based spatial RNA	DNA-nanoball array with ~500 nm coordinate spacing; whole-transcriptome, large field of view (59).	Panoramic mapping of lymph-node or extranodal architecture at high native spatial density.	Very high data volume, capture/binning choices, computational maturity, and tissue-processing requirements complicate routine FFPE translation.

Platform specifications evolve; manufacturer documentation in References (57–58, 60–61, 64–65) should be checked at study initiation and is used here only for current technical specifications or workflow options. Manufacturer sources are not evidence of biological validity, analytic superiority, or clinical utility; peer-reviewed studies should support performance and interpretation claims wherever available. Spatial support is not equivalent to biological resolution, and cell-contact claims require cell-resolved localization plus segmentation quality control. CODEX, co-detection by indexing; DLBCL, diffuse large B-cell lymphoma; DNA, deoxyribonucleic acid; FFPE, formalin-fixed paraffin-embedded; IMC, imaging mass cytometry; QC, quality control; RNA, ribonucleic acid; SMI, Spatial Molecular Imager.

Manufacturer documentation in References (57–58, 60–61, 64–65) is used only for time-sensitive platform specifications, such as nominal feature size, panel capacity, and current workflow options. Biological validity, analytic performance, and cross-platform interpretation are based on peer-reviewed studies wherever available; a manufacturer specification is not treated as evidence of biological resolution, clinical validity, or platform superiority.

Recent non-spatial studies have also nominated PLEKHG7 as a progression- and prognosis-associated program and a Limosilactobacillus reuteri-derived metabolite/AHR–mTOR axis as a modifier of chemotherapy response in DLBCL (66, 67). These reports broaden the candidate mechanism space, but because they do not preserve tissue coordinates, they are treated here as biology for future spatial validation rather than evidence of an established spatial niche.

This does not mean that lower-resolution platforms are unhelpful. Spot-based or regional transcriptomic assays can be powerful for discovering broad immune, stromal, metabolic, and tumor-state fields across larger tissue areas. Their limitation is interpretive, not scientific: they should be used to support regional density, co-localization, or pathway hypotheses rather than direct cell-cell contact claims. Conversely, cell-resolved platforms can support proximity and neighborhood analyses but may sacrifice transcriptome breadth or introduce segmentation bias. A strong validation program uses each platform for the estimand it can measure reliably.

Treatment resistance is dynamic, but longitudinal inference should follow a prespecified comparability hierarchy: Tier 1, the same lesion sampled with the same biopsy modality; Tier 2, a different lesion at the same anatomical site with matched biopsy modality and processing; and Tier 3, a different anatomical site or otherwise unmatched sample. Tier 1 provides the strongest basis for a paired temporal delta, Tier 2 supports a qualified within-site association, and Tier 3 is exploratory. All tiers should retain identical ROI hierarchy, physical scale, platform tier, marker definitions, and normalization. When these conditions are not met, changes should be described as associated with or consistent with treatment exposure, not as treatment-induced spatial remodeling, because apparent deltas may reflect lesion selection, anatomy, sampling, or technical variation.

The practical route to clinical translation is a two-step model. Discovery cohorts can use high-dimensional single-cell and spatial assays to nominate recurrent resistance modules. Validation cohorts should then test reduced, FFPE-compatible pathology assays with locked ROI definitions, scale choices, and sensitivity analyses. The goal is not to replace pathology but to extend it with reproducible spatial metrics that are interpretable across centers.

A proposed roadmap for future lymphoma spatial immunology studies is summarized in **Box 2**. This path also aligns better with routine hematopathology. Pathologists already recognize necrosis, crush artifact, vascularity, residual follicles, stromal septa, extranodal compartments, and tumor-rich regions. Spatial multi-omics should add quantifiable and reproducible measurements to this expertise rather than replace it. A clinically useful assay will likely be reduced-marker, FFPE-compatible, interpretable under the microscope, and robust across scanners, centers, and tissue-processing conditions.

Box 2 **Proposed roadmap for next-generation lymphoma spatial immunology studies.**

- Build prospective, treatment-annotated cohorts across R-CHOP, pola-R-CHP, CAR T-cell therapy, bispecific antibodies, ADCs, and targeted combinations.
- Treat anatomical site as a design factor before making genotype-to-niche claims; use site-matched contrasts or explicit rare-site fallback strategies.
- Convert discovery findings into FFPE-compatible, pathologist-anchored ROI or tile/bin metrics before relying on cell-level distances in dense or artifact-prone biopsies.
- Collect paired baseline, on-treatment, progression, and relapse tissue when feasible, and report temporal delta metrics only when site, lesion class, ROI hierarchy, and measurement support are comparable.
- Preserve coordinate-level spatial data, images, masks, scale factors, site labels, and analysis code sufficient to recompute reported neighborhood or interface metrics.
- Functionally test priority axes and spatial-pharmacology hypotheses in co-culture, organoid-like, tissue-explant, microfluidic, or *in vivo* systems that preserve relevant lymphoma microenvironment components.

Overall, current studies converge on recurrent spatial failure modes: antigen-presentation alteration, CD8+ T-cell dysfunction, regulatory immune enrichment, macrophage-rich suppressive hubs, stromal or vascular compartmentalization, and tumor-rich immune-excluded neighborhoods. These patterns justify prospective validation, but the manuscript intentionally preserves boundaries between observed tissue organization, computational inference, and experimentally proven mechanism. Detailed technical implementation, data-lineage classification, and platform-support rules are retained in **Supplementary Tables S6–S8** to keep the main article focused on clinical interpretation.

## Limitations

This Review has several limitations. First, it is a structured narrative synthesis rather than a preregistered systematic review; screening and evidence charting were not performed independently in duplicate, so study selection and classification remain susceptible to reviewer judgment. Second, restriction to English-language, peer-reviewed literature may introduce language and publication bias, and prioritization of high-resolution, clinically annotated, or higher-visibility studies may underrepresent negative or smaller datasets. Third, the core literature is heterogeneous in anatomical site, treatment setting, tissue handling, platform resolution, segmentation, and outcome annotation, which limits direct comparison and precludes quantitative pooling. Fourth, most evidence is cross-sectional or associative, and genuinely prospective treatment-by-biomarker validation is not established in the current core evidence base. Finally, platform specifications and the rapidly evolving 2024–2026 literature may change after the final search date. These limitations support cautious interpretation of the proposed genotype-to-niche and spatial-pharmacology models as testable frameworks rather than validated clinical algorithms.

## Conclusions

Single-cell and spatial multi-omics support an ecosystem-level view of large B-cell lymphoma in which malignant B-cell states, effector-cell dysfunction, regulatory and myeloid remodeling, stromal or vascular compartmentalization, and local communication programs jointly shape immune escape and therapy resistance. A niche-centered cancer-immunology framework can connect high-resolution biology with pathology-ready biomarkers and rational immunotherapy trial design, but prospective treatment-matched validation and functional testing are essential before these insights can guide patient management.

## Supplementary material

Supplementary Figure 1 Evidence structure supporting the DLBCL spatial framework. The figure relates the 16-study core evidence base to the nine representative studies in Main **Table 1**, shows that individual studies may contribute to multiple non-exclusive evidence streams, and distinguishes observation, computational prioritization, functional validation, and genuinely predictive evidence. Candidate communication axes remain hypotheses until functionally validated.

Supplementary Figure 2 Candidate spatial-pharmacology mechanisms and measurable proxies in DLBCL. The figure aligns ADC-containing regimens, CD20×CD3 bispecific antibodies, and CAR T-cell therapy with pathology-measurable spatial proxies and evidence caveats. Solid lines indicate direct observation or physical contact, dashed arrows indicate computational inference, and orange dashed frames indicate candidate mechanisms requiring dynamic or functional validation.

Supplementary Table 1 These tables provide study-level evidence charting, malignant B-cell, T-cell/regulatory, and myeloid/macrophage summaries, terminology and evidence-boundary auditing, data-lineage and coordinate-level reuse assessment, genotype-to-niche-to-metric hypotheses, rare-site fallback strategies, and pathology implementation rules. The main article remains complete and interpretable without the **Supplementary Information**.
